# Uncomplicated obesity is associated with abnormal aortic function assessed by cardiovascular magnetic resonance

**DOI:** 10.1186/1532-429X-10-10

**Published:** 2008-02-13

**Authors:** Monique R Robinson, Michaela Scheuermann-Freestone, Paul Leeson, Keith M Channon, Kieran Clarke, Stefan Neubauer, Frank Wiesmann

**Affiliations:** 1 Oxford Centre for Clinical Magnetic Resonance Research (OCMR), Department of Cardiovascular Medicine, University of Oxford, Oxford, UK; 2 Department of Physiology, Anatomy & Genetics, University of Oxford, Oxford, UK; 3 Department of Cardiovascular Medicine, University of Oxford, Oxford, UK; 4 University of Oxford Centre for Clinical Magnetic Resonance Research, Department of Cardiovascular Medicine, John Radcliffe Hospital, Headley Way, Oxford, OX3 9DU, UK

## Abstract

**Aims:**

Obese subjects with insulin resistance and hypertension have abnormal aortic elastic function, which may predispose them to the development of left ventricular dysfunction. We hypothesised that obesity, uncomplicated by other cardiovascular risk factors, is independently associated with aortic function.

**Methods and results:**

We used magnetic resonance imaging to measure aortic compliance, distensibility and stiffness index in 27 obese subjects (BMI 33 kg/m^2^) without insulin resistance and with normal cholesterol and blood pressure, and 12 controls (BMI 23 kg/m^2^). Obesity was associated with reduced aortic compliance (0.9 ± 0.1 vs. 1.5 ± 0.2 mm^2^/mmHg in controls, p < 0.02) and distensibility (3.3 ± 0.01 vs. 5.6 ± 0.01 mmHg^-1 ^× 10^-3^, p < 0.02), as well as higher stiffness index (3.4 ± 0.3 vs. 2.1 ± 0.1, p < 0.02). Body mass index and fat mass were negatively correlated with aortic function. Leptin was higher in obesity (8.9 ± 0.6 vs. 4.7 ± 0.6 ng/ml, p < 0.001) and also correlated with aortic measures. In multiple regression models, fat mass, leptin and body mass index were independent predictors of aortic function.

**Conclusion:**

Aortic elastic function is abnormal in obese subjects without other cardiovascular risk factors. These findings highlight the independent importance of obesity in the development of cardiovascular disease.

## Introduction

Obesity affects approximately 300 million people worldwide, and another 750 million are believed to be overweight [1], representing one of the largest health care challenges of our time. Obesity is associated with high levels of adiposity, significantly increased levels of adipokines such as leptin [2] and elevated levels of the inflammatory marker C-reactive protein (CRP) [3]. Landmark studies have linked obesity with a higher risk of developing heart failure [4].

Subjects with obesity have altered aortic function [3,5,6]. Physiologically, the aorta maintains low left ventricular after-load, promotes optimal sub-endocardial coronary blood flow [7], and transforms pulsatile into more laminar blood flow. Increased aortic stiffness leads to higher left ventricular systolic pressures, diminished sub-endocardial blood supply [7] and may ultimately contribute to left ventricular dysfunction [8,9]. These changes in arterial mechanics are also associated with coronary artery disease [10], hypertension [11,12], diabetes [13,14], and hypercholesterolaemia [15-17]; disorders which themselves are more common in obesity Therefore, it has been difficult to determine the *independent *effect of obesity on vascular function.

In this study, we employed the unique features of cardiovascular magnetic resonance imaging – direct visualisation of cardiac and aortic mechanics, with high temporal and spatial resolution, even in subjects with large subcutaneous thoracic fat deposits [18,19] – to test the hypothesis that obesity is independently associated with abnormal aortic function, in adults without confounding factors such as diabetes, insulin resistance, hypertension, or coronary artery disease.

## Methods

### Subjects

Control and obese subjects were recruited from the general population of Oxfordshire via newspaper advertisements. The study was approved by the local ethics committee, and subjects gave their informed consent prior to participation.

### Blood assays

Participants had fasting venous blood samples collected to assess hepatic and renal function, full blood count, lipid profile, insulin, glucose, C reactive protein (CRP) and leptin. Lipid profile was based on total cholesterol, high density lipoproteins (HDL), triglycerides and a calculated low density lipoprotein (LDL) level [20]. Leptin (LINCO Research Inc., St. Charles Missouri) and C reactive protein (CRP) (MP Biomedicals, Orangeburg, NY) were measured using commercially available ELISA techniques.

### Exclusion criteria

To investigate the independent effect of obesity on aortic function, we excluded patients with cardiovascular risk factors or factors that might contribute to sub-optimal vascular function. Hypertensives were identified and excluded based on the Joint National Council on Prevention, Detection, Evaluation and Treatment of High Blood Pressure definitions [21]. Diabetics were identified from medical history or fasting venous blood glucose level ≥ 6.7 mmol/L [22]. Furthermore, the homeostasis insulin model assessment (HOMA) formula was used to calculate an insulin resistance (IR) score [23]. Men with an IR score of > 2.35 and women with a score > 1.88 were excluded based on the European Group for the study of Insulin Resistance (EGIR) guidelines [24]. Smokers, subjects with a history of cerebrovascular or coronary artery disease, those with total blood cholesterol levels > 6 mmol/L and those with abnormal renal, hepatic or haematological function were not included. Additionally, those with contraindications to CMR were not recruited.

### Assessment of body size

All participants were weighed on an electronic Seca scale and height was measured on an adjustable Seca standing stadiometer. These measures were used to calculate body mass index. Waist and hip circumferences were measured using a tape measure. Bioelectric impedance using the Bodystat^® ^1500 was used to assess fat mass.

### Cardiovascular magnetic resonance imaging

**Figure 1:**
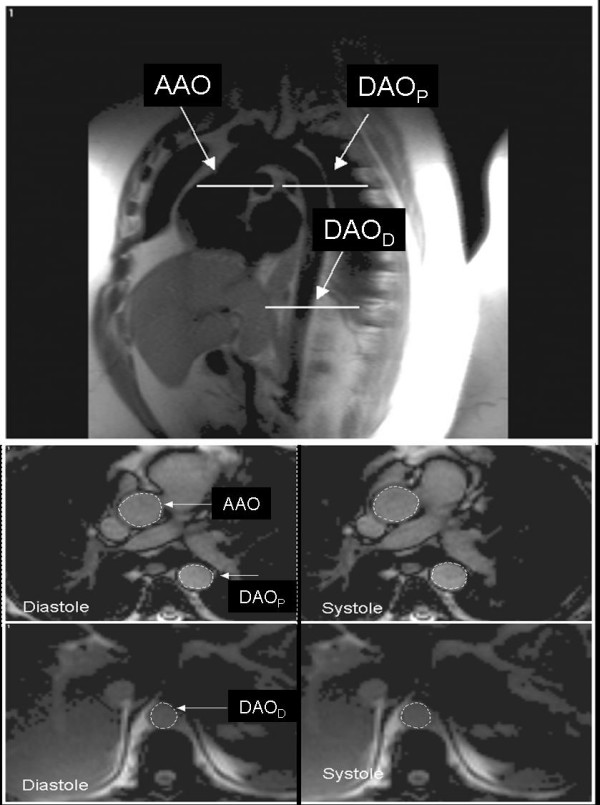
**CMR image in coronal-sagittal orientation indicating measurement levels in the aorta (a).** AAO indicates ascending aorta; DAO_P_, proximal descending aorta; DAO_D_, distal descending aorta. Transverse CMR images demonstrating the ascending and proximal descending aorta (b, c) and the distal descending aorta (d, e) in systole and diastole.

CMR studies were performed on a 1.5 Tesla clinical MR system (Siemens Sonata, Erlangen, Germany) as previously described [25]. For aortic imaging, a 2-element array surface coil on the chest was combined with a spine-coil array. Aortic indices were assessed using TrueFISP cine sequences with the following parameters: TR/TE 2.8 ms/1.4 ms and 15 lines per phase with a temporal resolution of 24 frames per second. Sampling bandwidth was 930 Hz/pixel with a matrix of 192 × 118 over a FoV of 380 × 332 mm, resulting in an in-plane resolution of 1.97 × 2.81 mm. Aortic cine images were acquired in two transverse planes, based on sagittal-oblique pilots (Figure 1a): at the pulmonary arch for the ascending and descending aorta and 10 cm below the diaphragm for the distal descending aorta (Figure 1b and 1c). All participants had their resting blood pressure taken immediately before the cardiac magnetic resonance study. For cardiac analysis localiser images were acquired followed by vertical long axis (VLA) and horizontal long axis (HLA) cine images. A short axis stack of contiguous images was then acquired (slice thickness 7 mm, inter-slice gap 3 mm).

Aortic cross-sections were manually contoured using CMR Tools^® ^(Imperial College, London, UK). Vascular compliance, distensibility and stiffness index were calculated as described previously [18]. Aortic compliance is the absolute change in area per unit of pressure whereas distensibility is the relative change per unit pressure. Stiffness index examines the logarithmic relationship between pressure and the relative change in aortic cross-sectional area. This takes into account the variation in background arterial distending pressure. Mean aortic compliance, distensibility and stiffness index were calculated by averaging the regional measures. Left ventricular volumes and mass were obtained from the short axis stack by manually contouring end-diastolic and end-systolic endocardial and epicardial borders from base to apex, using Siemens analytical software (ARGUS^©^). Left ventricular end-diastolic volume (EDV), end-systolic volume (ESV), left ventricular mass (LVM), ejection fraction (EF), stroke volume (SV) and cardiac output (CO) were calculated and, where appropriate, normalised for body size.

### Statistical analysis

Statistical analysis was carried out using SPSS 11.0. Arterial compliance, distensibility, stiffness index and myocardial mass were not normally distributed and were investigated using non-parametric Mann-Whitney test and Spearman's analysis for correlation. All values are reported as mean ± the standard error of the mean (SEM) and a *p *value of < 0.05 was considered significant. Multiple linear regression was carried out correcting for gender and height to determine predictors of aortic function.

## Results

Demographic characteristics of the study groups are in Table 1. Ages and lean mass of obese and lean subjects were not different. The obese group were shorter, with a 1.4 times higher BMI (p < 0.01) and 1.9 times higher fat mass (p < 0.01). There was no significant difference between groups in waist-hip ratio, systolic (SBP) or diastolic blood pressure (DBP) or mean arterial pressure.

**Table 1:** Demographic data-obese and control group

	**Control Subjects**	**Obese Subjects**
Sample size	12	27
Age (y)	53 ± 10	49 ± 11
Male: Female	8:4	13:14
Weight (kg)	75.0 ± 12.6	98.3 ± 19.7†
Height (m)	1.8 ± 0.01	1.70 ± 0.01*
BMI (kg/m2)	23.9 ± 2.7	33.8 ± 3.0†
Fat Mass (kg)	20.2 ± 6.8	38.3 ± 11.9†
Lean Mass (kg)	54.0 ± 14.9	57.6 ± 14.5
WC (cm)	86 ± 8	113 ± 15 *
WHR	0.9 ± 0.01	1.0 ± 0.01
SBP (mmHg)	127 ± 10	130 ± 9
DBP (mmHg)	76 ± 9	80 ± 8

BMI, body mass index; SBP, systolic blood pressure; DBP, diastolic blood pressure; MAP, mean arterial pressure; WC, waist circumference; WHR, waist hip ratio. *p < 0.05, †*p *< 0.01. Data are presented as means ± standard deviation.

### Blood parameters (Table 2)

**Table 2:** Biochemical assays-obese and control groups

	**Control Subjects**	**Obese Subjects**
Total Cholesterol (mmol/L)	4.94 ± 0.12	4.93 ± 0.15
HDL Cholesterol (mmol/L)	1.53 ± 0.1	1.17 ± 0.05*
LDL Cholesterol (mmol/L)	2.95 ± 0.20	3.09 ± 0.21
Triglycerides (mmol/L)	1.00 ± 0.11	1.31 ± 0.12*
Fasting Glucose (mmol/L)	5.07 ± 0.09	5.0 ± 0.11
Insulin (μmol/L)	2.91 ± 0.33	4.69 ± 0.56*
HOMA	0.61 ± 0.09	0.65 ± 0.12
Leptin (ng/ml)	4.69 ± 0.57	8.98 ± 0.58†
CRP (mg/L)	3.47 ± 0.47	5.26 ± 0.56

HOMA, homeostasis insulin model assessment; CRP, C reactive protein. *p < 0.05, †*p *< 0.01. Data are presented as means ± standard error of the mean.

There was no significant difference in total or LDL cholesterol levels between groups. Triglyceride levels were 31% higher in the obese (p < 0.05) and HDL levels 13% lower (p < 0.05). Leptin levels were 91% higher in the obese (p < 0.01) whereas CRP levels were not significantly different. Fasting glucose levels were similar between groups. Insulin levels were higher in the obese, but HOMA insulin resistance scores did not vary. BMI correlated positively with leptin (*r *= 0.8, *p *< 0.001) and CRP (*r *= 0.6, *p *< 0.001) and negatively with HDL (*r *= -0.7, *p *< 0.001). Fat mass showed positive correlations with leptin (*r *= 0.8, *p *< 0.001) and CRP (*r *= 0.5, *p *= 0.001), and negative correlation with HDL cholesterol (*r *= -0.5, *p *< 0.001).

### Left ventricular function

There was no difference in ejection fraction between groups. The control cohort was taller and had larger cardiac volumes. However, there were no significant differences between cohorts in SV, EDV, ESV, and LVM indexed for height (Table 3).

**Table 3:** Left ventricular function-obese and control groups

	**Control Subjects**	**Obese Subjects**	***P *value**
EF (%)	65 ± 2	63 ± 3	0.7
ESV index (ml/m)	28 ± 2	26 ± 1	0.5
EDV index (ml/m)	81 ± 5	70 ± 3	0.1
SV index (ml/m)	53 ± 4	45 ± 3	0.1
LVM index (g/m)	81 ± 6	87 ± 4	0.4

Data are presented as mean ± standard error of the mean. All data represent height indexed values. ESV, end-systolic volume; EDV, end-diastolic volume;SV, stroke volume; EF, ejection fraction, LVM, left ventricular mass.

### Aortic function and obesity (Table 4)

**Table 4:** Regional aortic elastic function – obese and control groups

	**Control Subjects**	**Obese Subjects**
AAO Compliance (mm^2^/mmHg)	2.04 ± 0.23	1.69 ± 0.26
AAO Distensibility (mmHg^-1 ^× 10^-3^)	3.60 ± 0.44	3.30 ± 0.63
AAO Stiffness index	2.33 ± 0.1	2.55 ± 0.2
DAO_P _Compliance (mm^2^/mmHg)	1.40 ± 0.17	0.83 ± 0.15*
DAO_P _Distensibility (mmHg^-1 ^× 10^-3^)	5.00 ± 0.7	3.20 ± 0.5^†^
DAO_P _Stiffness index	2.06 ± 0.1	2.89 ± 0.3
DAO_D _Compliance (mm^2^/mmHg)	0.93 ± 0.18	0.56 ± 0.12^‡^
DAO_D _Distensibility (mmHg^-1 ^× 10^-3^)	8.10 ± 0.3	3.60 ± 0.7*
DAO_D _Stiffness index	1.89 ± 0.3	4.00 ± 0.6^‡^

AAO, ascending aorta; DAO_P_, proximal descending thoracic aorta;

DAO_D_, descending (abdominal) aorta. Data are presented as mean ± standard error of the mean. *p = 0.02, ^† ^*p *= 0.03, ^‡ ^*p *= 0.04

Obesity was associated with a significant reduction in compliance in the proximal descending thoracic aorta and the distal descending abdominal aorta (Figure 1). Furthermore, there was a corresponding decrease in distensibility of the proximal descending aorta and distal descending aorta. Stiffness index (*β*) was significantly higher in the obese at the level of the distal descending aorta only. There was no significant difference between groups in aortic compliance, distensibility or stiffness index in the ascending aorta.

**Figure 2:**
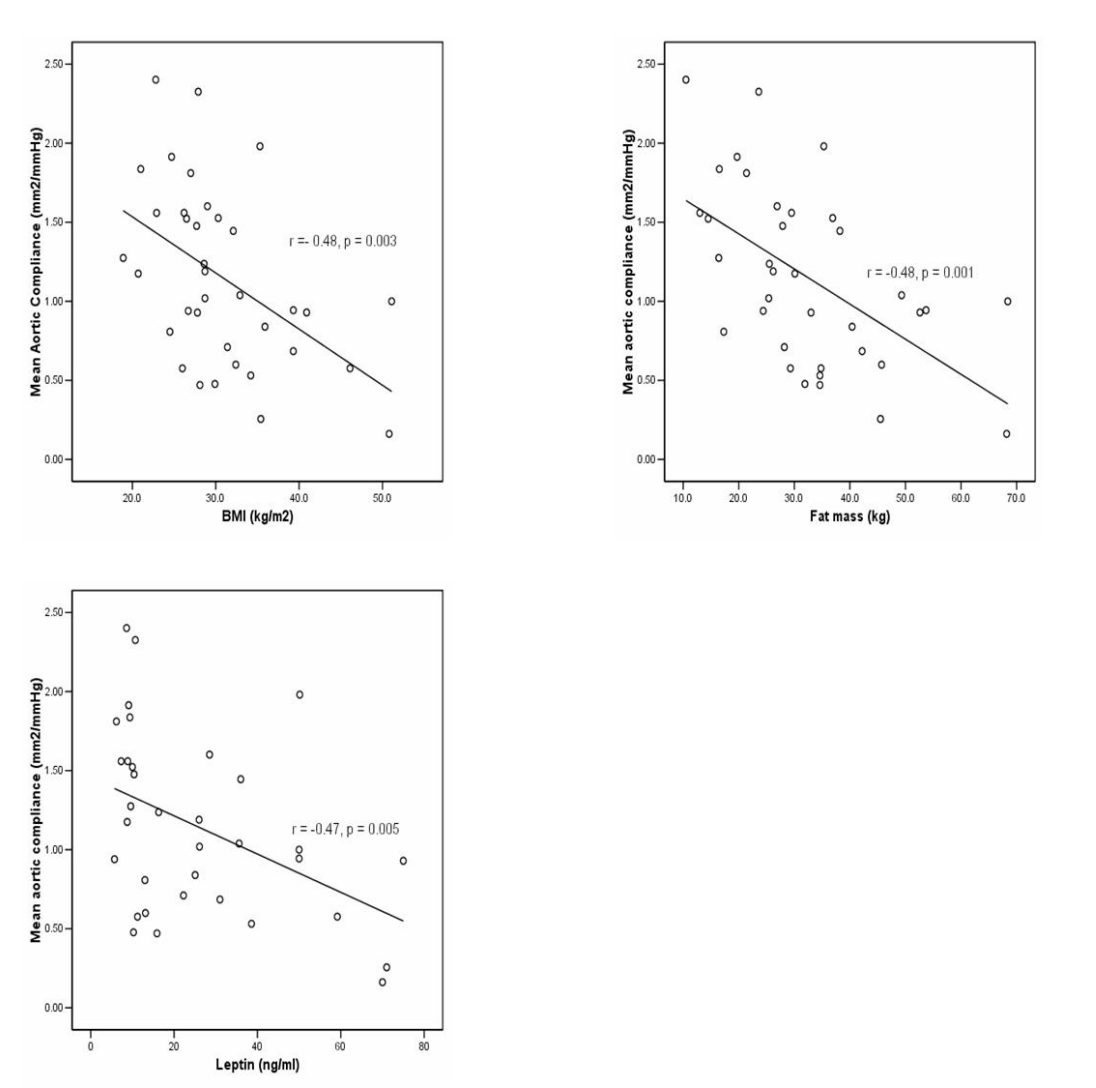
Mean aortic compliance had a negative correlation with (a) body mass index (BMI), (b) fat mass and (c) leptin.

**Figure 3:**
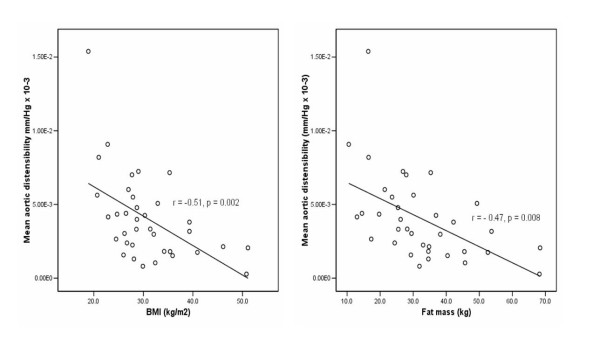
Mean aortic distensibility correlated negatively with (a) body mass index (BMI) and (b) fat mass.

Conventional indicators of obesity were significantly correlated with aortic function. Mean aortic compliance in the obese was 40% lower (0.99 ± 0.11 vs. 1.45 ± 0.15 mm^2^/mmHg, *p *= 0.021) and distensibility 59% lower (3.3 ± 0.004 vs. 5.6 ± 0.001 mmHg^-1 ^× 10^-3^, *p *= 0.023). Compliance showed significant negative correlations with BMI (*r *= -0.48 *p = *0.003), fat mass (*r *= -0.55, p = 0.001), and leptin (*r *= -0.47, *p *= 0.005) (Figure 2) and significant positive correlations with HDL (*r *= 0.66, *p *< 0.001). Aortic distensibility correlated negatively with BMI (*r *= -0.51, *p *= 0.002) (Figure 3a), and fat mass (*r *= -0.58, *p *< 0.001) (Figure 3b). HDL showed a significant positive correlation with distensibility (*r *= 0.47, *p *= 0.008).

There was also a negative correlation noted between waist circumference and aortic compliance (r = -0.53, p = 0.001) and distensibility (r = -0.56, p = <0.001). There were no significant correlations between waist-hip ratios and measures of aortic compliance (r = -0.06, p = 0.75) or distensibility (r = -0.06, p = 0.75).

### Determinants of aortic function

Using independent multiple linear regression analysis, a significant negative linear relationship was found between aortic compliance and leptin (Regression Coefficient (RC) = -0.017, 95% Confidence Intervals (CI) = -0.027 to -0.007 *p *= 0.002), fat mass (RC = -0.029, 95% CI = -0.042 to -0.015, *p *< 0.001), and BMI (RC = -0.045, 95%CI = -0.70 to -0.020, *p *= 0.001). On the basis of adjusted r square analysis, fat mass emerged as the strongest predictor of aortic compliance. A significant positive association was noted between aortic compliance and HDL cholesterol (RC = 0.873, 95%CI = 0.278 to 1.468, *p *= 0.006). Distensibility was similarly determined by fat mass (RC = -0.041, 95%CI = -0.057 to -0.026, *p *< 0.001), BMI (RC = -0.060, 95%CI = -0.09 to -0.03, p < 0.001) and leptin (RC = -0.019 × 10^-2^, 95%CI = -0.32 to -0.005, p = 0.008). HDL (RC = 1.309, 95%CI = 0.596 to 2.020, *p *= 0.001) was positively associated with distensibility. There was no significant relationship demonstrated between CRP, insulin or insulin resistance and aortic function. Thus, fat mass, leptin and BMI emerged as the main predictors of abnormal aortic function in this population.

## Discussion

In this study, cardiovascular magnetic resonance imaging revealed significant changes in aortic mechanical function in an obese population without hypertension, diabetes, insulin resistance or hypercholesterolaemia. The descending aorta had significantly lower compliance, distensibility and a higher stiffness index – all indicators of decreased mechanical and intrinsic elastic function. This functional abnormality strongly correlated with BMI, fat mass, leptin, waist circumference and HDL levels. Even after adjustment for the potential confounders of gender and height, fat mass emerged as the strongest predictor of decreased aortic elasticity, closely followed by leptin, BMI and HDL.

Previous studies of obesity have been largely limited to peripheral vessels, and usually examined obese cohorts with concomitant insulin resistance [26,27], diabetes [27-29], hypercholesterolaemia [27] and hypertension [30,31]. Furthermore, conflicting results on the relationship between increasing BMI, adiposity and vascular stiffness have been published. Oren *et al *[30] reported *increased *aortic compliance in obese subjects compared with lean controls and Raison et al [32] demonstrated reduced vascular peripheral resistance in obesity. More recent studies have evaluated arterial distensibility in peripheral vessels [3] and pulse wave velocities [33] and suggest a *negative *correlation between fat mass and aortic compliance, more consistent with an adverse impact of obesity on the vasculature.

Oren *et al *used diastolic blood pressure decay and pulse pressure relative to stroke volume as surrogate measures of compliance of the whole aorta. These were measured by placing a pressure catheter in the ascending aorta. Magnetic resonance imaging has the advantage of studying changes in aortic compliance in different segments of the aorta. Using CMR, Danias *et al *[5] studied the ascending aorta in an obese population with cardiac risk factors and reported no difference in compliance compared to controls. However, they did find a reduction in elasticity of the abdominal aorta. They hypothesised that the changes may have been due to physical compression by abdominal fat or structural changes in the vessel wall. As the study included subjects with cardiac risk factors these may also have independently influenced vascular function. Our study demonstrates that changes in distensibility occur in the descending thoracic aorta as well as the abdominal aorta and are independent of abdominal size. These findings suggest the changes in aortic function are less likely to be due to physical compression from abdominal fat. Furthermore, our cohort did not have cardiac risk factors, which suggests obesity has an independent impact on vascular function.

Similar to Danias *et al *[5] we found no change in function in the proximal aorta. The precise reason for the proximal sparing of the vessel remains unclear. It is possible that aortic dysfunction in obesity begins distally with an ascending pattern of progression. The aorta is a physiologically heterogeneous vessel with elastin:collagen ratios decreasing distally along its length. Regions with higher proportions of elastin have physiologically greater abilities to stretch and recoil. Impairment of vascular elasticity might commence in vessel sections physiologically less compliant [34], and this might then affect the entire arterial tree if obesity is sustained.

Although we excluded all subjects with raised glucose or insulin resistance, our population was hyperinsulinaemic. In work done by Ferrannini *et al *[35], it was recognised that although insulin hypersecretion can occur in adults with uncomplicated obesity, the prevalence of insulin resistance is low. Further, it was suggested that in the obese with no evidence of insulin resistance, the risk for the development of cardiovascular disease might differ from that seen in the insulin resistant patient. Additionally work done during the San Antonio Heart Study [36] demonstrated that during an eight year prospective trial, subjects with high HOMA scores (i.e. with evidence of insulin resistance) were the ones at highest risk for cardiovascular events.

The reduced aortic compliance and distensibility seen in individuals with uncomplicated obesity was unrelated to the inflammatory status, as CRP was not correlated to aortic function. Anthropometric parameters and leptin were the strongest predictors of aortic function and therefore may be more important in the pathogenesis of early aortic disease. Elevated leptin has been shown to increase atherosclerotic risk [37,38]. Knudson *et al *[39] demonstrated the presence of leptin receptors on coronary artery endothelium and that through increased endothelial oxidative stress hyperleptinaemia resulted in significant arterial endothelial dysfunction. Additionally, Zarkesh-Esfanai *et al *[40] have demonstrated that high leptin levels may lead to the activation of tumour necrosis factor alpha (TNFα). TNFα has been shown to decrease eNOS production and consequently increase vascular tone [41]. We have not measured TNFα but it is conceivable that chronically elevated leptin levels indirectly impair vascular elastic function via TNFα.

Abnormal aortic function is an independent predictor of the development of coronary artery disease and stroke [42], as well as left ventricular dysfunction. Interestingly, cardiac changes are not yet evident in our cohort with obesity despite a mean age of forty nine. The development of cardiac dysfunction may have been delayed by the absence of other risk factors or the selection of subjects with uncomplicated obesity has identified a specific group with adaptive processes that compensate for changes in aortic function. It would be of interest to determine whether cardiovascular disease and risk factors in obese individuals predisposes them to further decline in aortic function and determine how aortic dysfunction progresses over time in uncomplicated obesity.

Our study is limited by a relatively small sample size and these findings need to be investigated further in larger cohorts with uncomplicated obesity. The lack of variation in left ventricular function between the obese and lean subjects has been demonstrated in other studies [43]. However, with larger sample numbers to facilitate gender and obesity subgroup analysis on the basis of increasing BMI, a pattern towards worsening left ventricular function might have been noted. As changes in aortic distensibility are seen so early, it is possible that genetic factors are relevant to changes in aortic distensibility in obesity. Data on family history of cardiovascular disease was not available in our cohort and more detailed work will be required to investigate the possible contribution of inherited factors. Fat mass distribution is of interest to risk of cardiovascular disease [44] and can be assessed with magnetic resonance imaging. Future magnetic resonance research could incorporate these measures to determine how adiposity distribution contributes to changes in aortic function. This research could also study other indices of aortic function such as pulse wave velocity and more refined assessments of blood pressure, including use of central aortic pressure. As leptin is produced predominantly in adipocytes, a reduction in fat mass, rather than absolute weight reduction, might be more efficacious in restoring normal aortic function in this group of patients.

CMR is an excellent imaging modality for non-invasive quantitative assessment of vascular mechanics in a clinical study setting, but might prove impractical for screening for increased aortic stiffness in the general obese population. Our study suggests fat mass and BMI have a predictive potential for central arterial dysfunction. Unlike HDL and leptin measurements, which, though predictive, necessitate venepuncture and laboratory testing, BMI and fat mass are both easily measured with scales, callipers or bioelectric impedance. Earlier appreciation of the vascular risk posed by uncomplicated obesity encourages earlier and more aggressive treatment, thus reducing the morbidity and mortality associated with excess body weights.

## Authors' contributions

MRR carried out the study, analysed the data and prepared the manuscript. MS-F helped carry out the study. PL contributed to data analysis and prepared the manuscript. KMC contributed to the manuscript preparation. KC contributed to the study design and manuscript preparation. SN contributed to the study design, data analysis and manuscript preparation. FW contributed to the study design and supervised the study.
